# Second-Line Treatment of Her2-Positive Metastatic Breast Cancer: Trastuzumab beyond Progression or Lapatinib? A Population Based Cohort Study

**DOI:** 10.1371/journal.pone.0138229

**Published:** 2015-09-16

**Authors:** Ariel Hammerman, Sari Greenberg-Dotan, Ilan Feldhamer, Haim Bitterman, Rinat Yerushalmi

**Affiliations:** 1 Chief Physician’s Office, Clalit Health Services Headquarters, Tel-Aviv, Israel; 2 The Bruce and Ruth Rappaport Faculty of Medicine, Technion–Israel Institute of Technology, Haifa, Israel; 3 Institute of Oncology, Davidoff Cancer Center, Beilinson Hospital, Rabin Medical Center, Petah Tikva, Israel; 4 The Sackler Faculty of Medicine, Tel-Aviv University, Tel-Aviv, Israel; Instituti Ospitalieri di Cremona, ITALY

## Abstract

**Background:**

The relative efficacy of lapatinib vs. continuing trastuzumab beyond progression (TBP) in HER2-positive metastatic breast cancer (MBC) patients, who progressed on first-line trastuzumab, is still unclear. The objective of this population based cohort study was to compare outcomes of lapatinib vs. TBP in daily practice.

**Methods:**

All HER2-positive MBC patients who began second-line anti HER2 therapy between 1st January 2010 and 30th August 2013 were selected from Clalit Health Services’ (CHS) electronic database. Available data on patient and disease characteristics and treatments were analyzed. The primary endpoint was overall survival (OS). Outcomes were compared using the Kaplan-Meier (log-rank) method and Cox proportional hazards model.

**Results:**

64 patients received second-line lapatinib and 93 TBP. The two treatment groups were similar in age and co-morbidity rates, but differed in proportion of prior adjuvant trastuzumab (lapatinib: 29.7%, TBP: 16.1%, *P* = 0.043) and rates of prior brain metastases (lapatinib: 32.8%, TBP: 10.8%, *P* = 0.01). Lapatinib median OS was 13.0 months (95% CI: 9.5–16.5) vs. 31.0 for TBP (95% CI: 20.6–41.4), *P*<0.001. On multivariate analysis, longer OS was preserved for TBP, after controlling for differences in age, adjuvant trastuzumab, duration of first-line trastuzumab therapy, brain metastases, visceral metastases and hormonal treatment [Hazard Ratio (HR) = 0.63, 95% CI: 0.40–0.99, *P* = 0.045].

**Conclusion:**

In this comparative cohort study, OS of HER2-positive MBC patients treated with TBP was significantly longer than with lapatinib. These results might be especially relevant in settings where ado-trastuzumab-emtansine (TDM-1), the current preferred agent in this setting, is not available yet for patients.

## Introduction

Metastatic breast cancer (MBC) is the leading cause of death from cancer among women worldwide. Approximately 15% to 20% of patients with breast cancer have tumors that over-express the human epidermal growth factor receptor-2 (HER2) protein. Prior to the era of HER2-targeted therapy, HER2-positive breast cancer was characterized by poor prognosis [1, 2]. Treatment of HER2-positive MBC has undergone substantial progress with the development of biological treatment options to block HER2 signaling pathways.

Trastuzumab, a recombinant, humanized, monoclonal antibody that binds to the extracellular domain of the HER2 protein, has been the standard of care for first-line treatment of HER2-positive MBC for more than a decade [3]. One phase III randomized study [4] and other retrospective analyses [5, 6] have shown that in patients who developed disease progression while on trastuzumab-containing therapy, treatment with trastuzumab 'beyond progression' (TBP) together with chemotherapy is feasible, safe and associated with improved clinical outcomes.

Lapatinib, a dual tyrosine kinase inhibitor that inhibits the HER2 receptor pathway and the epidermal growth factor receptor (EGFR), is an additional anti-HER2 therapeutic option for second-line, HER2-positive, MBC patients. Lapatinib's initial FDA approval in 2007 was for use in combination with capecitabine for the treatment of patients with HER2-overexpressing MBC who had received prior therapy including an anthracycline, a taxane, and trastuzumab [7].

Current ASCO practice guidelines [8] and NCCN guidelines [9] recommend continuation of HER2 blockade for patients with HER2-positive MBC that progresses on first-line trastuzumab-containing regimens, preferably with the newly available antibody-drug conjugate ado-trastuzumab emtansine (T-DM1). However, in settings where TDM-1 is not yet available, there is still a question whether trastuzumab should remain the HER2-suppressing agent throughout multiple lines of treatment or whether clinical benefit would be increased if a different HER2-targeted agent was used. Currently, no evidence-based data are available to help decide which of these two treatment strategies is preferable. Therefore, the objective of this study was to compare actual outcomes of second-line therapy of HER2-positive MBC patients, treated with either lapatinib or TBP.

## Patients and Methods

Clalit Health Services (CHS) is the largest healthcare provider organization in Israel, with over 4 million members, serving nearly 53% of the total population. Both TBP and second-line lapatinib are available and reimbursed in Israel since January 2010.

Using CHS' computerized population-based databases, we identified and compared outcomes of all HER2-positive MBC patients that started second-line anti-HER2 therapy with either lapatinib or TBP, after a first-line protocol containing trastuzumab, between January 1, 2010 and August 31, 2013.The cutoff date for data collection on patient treatments and outcomes was July 31, 2014.

The primary study end-point was overall survival (OS), defined as the interval between the initiation of second-line anti-HER2 therapy and death.

The following data were available from CHS' databases: age, Charlson co-morbidity index [10, 11] cancer diagnosis, all reimbursed anti-cancer drugs provided (in the early and metastatic stages of disease) sites of metastasis, and date of death. As no patient in the study cohort left CHS during the follow up period, we were able to retrieve full data on all the above variables for all patients. Other relevant clinical information, such as patient’s performance status, histopathological characteristics, ER/PR status, CT imaging results and compassionate or study treatments provided in later lines of therapy, were not available from the database. Since tamoxifen and aromatase inhibitors are fully reimbursed in Israel for cancer patients, use of hormonal treatment at any disease stage served us as a close proxy for ER/PR status.

The study was approved by the Clalit Health Services' Community Institutional Review Board (IRB approval # 0032-13-COM). As data was extracted from the existing computerized data bases of Clalit Health Services, the IRB waved the need for the patients to sign informed consent for the study. Patient records and information were anonymized and de-identified prior to analysis.

### Statistics

Kaplan-Meier estimate was used to compare OS using the log-rank test. Categorical variables were compared between the groups by chi-square tests. Continuous variables were compared by *t*-tests when normally distributed or by Mann-Whitney tests otherwise. *P*<0.05 was regarded as statistically significant. Cox proportional hazard regression model was used to adjust for the following potential prognostic confounders: age (under 50 and ≥ 50), hormonal therapy (yes/no), prior adjuvant treatment with trastuzumab (yes/no), duration of first-line metastatic trastuzumab therapy, brain metastases prior to second-line therapy (yes/no) and visceral metastases prior to second-line therapy (yes/no). Statistical analysis was performed using the Statistical Package for the Social Sciences (SPSS) software, version 20.0 (Chicago, IL, USA).

## Results

In total, 175 patients who had HER2-positive MBC were identified. Patients who did not receive trastuzumab in their first-line protocol (n = 18) were excluded from this analysis. Therefore, the final study population included 157 patients; 64 treated with lapatinib and 93 with TBP (S1 Data). The groups were found to be balanced in most variables available from the CHS' database (Table 1), apart from a higher proportion of patients in the lapatinib group that had received prior adjuvant trastuzumab (29.7% vs. 16.1%, *P* = 0.043) and more patients in the lapatinib group that had brain metastases at initiation of second-line treatment (32.8% vs. 10.8%, *P* = 0.001). Median follow up time was 16.0 months. All 64 patients on lapatinib were concurrently treated with capecitabine. Ninety one percent (58 patients) received capecitabine for the whole duration of lapatinib therapy. The 93 patients treated with second-line trastuzumab received a variety of parallel therapies, 80% (74 patients) were treated concurrently with chemotherapy and 20% (19 patients) with hormonal therapy (Table 2).

**Table 1:** Baseline demographics and patient clinical characteristics.

Characteristic	Lapatinib (*N* = 64)	TBP (*N* = 93)	*P*-value
Age (years)	58.5± 13.2	57.6± 13.7	NS^a^
Charlson comorbidity score (average)	3.39	3.10	NS^b^
Duration of first-line metastatic trastuzumab treatment (average, months)	11.4± 10.2	12.1± 11.1	NS^b^
Prior adjuvant trastuzumab	19 (29.7%)	15 (16.1%)	**0.043** ^c^
Time to relapse after completion of adjuvant trastuzumab (average, months)	15.7± 15.0	19.3± 17.9	NS^a^
Hormonal treatment at any disease stage	28 (43.8%)	53 (57.0%)	NS^c^
Brain metastases	21 (32.8%)	10 (10.8%)	**0.001** ^c^
Prior WBR*	18 (86%)	10 (100%)	NS^c^
Visceral metastases	38 (59.4%)	49 (52.7%)	NS^c^

^a^t-test

^b^Mann-Whitney test

^c^chi-square test

*WBR- Whole Brain Irradiation

**Table 2:** Drugs administered in parallel with second-line anti Her-2 therapy.

	Drugs administered with Lapatinib (*N* = 64)	Drugs administered with Trastuzumab (*N* = 93)
Letrozole		9 (9.6%)
Exemestane		5 (5.4%)
Anastrozole		3 (3.2%)
Fulvestrant		2 (2.2%)
**Hormonal therapy (total)**	**0 (0%)**	**19 (20.4%)**
Vinorelbine		40 (43.0%)
Paclitaxel		21 (22.6%)
Docetaxel		2 (2.2%)
Gemcitabine		4 (4.3%)
5FU+ cisplatin		1 (1.1%)
Capecitabine	64 (100%)	6 (6.5%)
**Chemotherapy (total)**	**64 (100%)**	**74 (79.6%)**

There were altogether 87 deaths: 44 (68.8%) in the lapatinib group and 43 (46.2%) in the trastuzumab group. Median OS was 13.0 months for lapatinib (95% CI: 9.5–16.5) and 31.0 months for TBP patients (95% CI: 20.6–41.4) (Table 3). On multivariate analysis, the adjusted OS hazard ratio (HR) was 0.63 (95% CI: 0.40–0.99, *P* = 0.045), in favor of TBP (Table 4). Hormonal treatment (at any stage of the disease) was found to be associated with a significantly better outcome (HR = 0.54, *P* = 0.006).

**Table 3:** Second-line lapatinib vs. trastuzumab univariate analysis.

Variable	Overall survival	
	Lapatinib (*N* = 64)	TBP (*N* = 93)
Events, n (%)	44 (69%)	43 (46%)
Median, months (95% CI)	13.0 (9.5–16.5)	31.0 (20.6–41.4)

**Table 4:** Overall survival hazard ratio, multivariate Cox regression analysis.

Effect	Hazard ratio	95% CI	*P* value
TBP vs. second-line lapatinib	0.63	0.40–0.99	**0.045**
Age ≥ 50 vs. <50	1.65	0.97–2.80	0.064
Hormonal treatment*(yes vs. no)	0.54	0.34–0.84	**0.006**
Prior adjuvant trastuzumab (yes vs. no)	1.19	0.70–2.02	0.511
Duration of first-line metastatic trastuzumabtreatment (months)	0.96	0.94–0.99	**0.006**
Brain metastases(yes vs. no)	1.53	0.88–2.69	0.136
Visceral metastases(yes vs. no)	1.33	0.86–2.04	0.204

* At any stage of the disease treatment

## Discussion

This retrospective cohort study suggests a significantly better survival outcome in HER2-positive MBC patients treated with TBP, rather than with lapatinib, as an anti-HER2 backbone for second-line MBC therapy. The study's two treatment groups were found to be comparable in age and rate of comorbidities. However, two main between-group differences may have affected outcomes. Patients in the lapatinib group were significantly more likely to have received adjuvant trastuzumab and more patients had prior brain metastases. This might suggest that patients in the lapatinib group initially presented with a more aggressive disease and despite prior adjuvant trastuzumab, the disease recurred. Nevertheless, in the multivariate analysis that controlled also for these differences (Table 3), the HR for OS was still significantly in favor of TBP-treated patients. The preferable outcomes for TBP patients occurred despite the assumed potential activity of lapatinib in patients with brain metastases [12].

Regulatory approval for lapatinib as a second-line treatment of MBC patients following prior therapy with trastuzumab was based on a phase III study which compared capecitabine alone against capecitabine plus lapatinib. The median time to progression in the combination arm was 8 months vs. 4 months in the capecitabine monotherapy arm (HR = 0.49, *P* < 0.001). Median OS was 17 and 14 months, respectively (HR = 0.87; *P* = 0.210) [13, 14].

TBP treatment benefit was demonstrated in one phase III study. Von Minckwitz et al. [4] randomized patients with HER2-positive MBC who had progressed on trastuzumab, to either capecitabine plus (continued) trastuzumab, or capecitabine monotherapy. Median time to progression was 8.2 months in the combination arm vs. 5.6 months in the capecitabine monotherapy arm (HR = 0.69; *P* = 0.04). Median OS was 26 versus 20 months (HR = 0.76; *P* = 0.26), respectively. Although the study did not reach its target accrual and has been criticized for its low power, it has still shown a benefit for TBP in terms of time to progression, its primary endpoint. It was hypothesized that trastuzumab may act as a chemotherapy sensitizer, and when a new cytotoxic is added, it confers additional benefit over the chemotherapeutic agent alone [15].

CEREBEL [16] was the first published prospective, phase III, head-to-head comparison study of lapatinib and trastuzumab in metastatic patients. Patients with brain metastases were excluded since the study's main objective was to evaluate the effect of the lapatinib-capecitabine therapy on the incidence of CNS as first site of relapse. CEREBEL was terminated early after analysis of interim safety and efficacy data. In the ITT population, median progression free survival (PFS) was shorter for the lapatinib-capecitabine arm (6.6 months) compared with trastuzumab-capecitabine (8.1 months); (HR = 1.30; 95% CI: 1.04–1.64, *P* = 0.021).

A major difference between the CEREBEL trial and the design of the current study was that prior trastuzumab for MBC was allowed in CEREBEL, but not required. In fact, only 35% of the CEREBEL patients received prior trastuzumab in the metastatic setting.

In contrast to the aforementioned randomized trials, this current retrospective, cohort study investigated the practical question of whether to prefer a TBP regimen or to switch to another anti-HER2 agent, lapatinib, in the second-line setting in the daily practice. This study presents 'real-life' outcomes of patients who faced failure on first-line trastuzumab and chemotherapy, including in patients who had already developed CNS involvement.

The results of the current study seem to be in line with the above CEREBEL trial results and with other increasing evidence showing less preferable outcomes with lapatinib compared to trastuzumab in various breast cancer settings. Recently, Gelmon et al. [17] have reported final results of the COMPLETE phase III study, comparing trastuzumab-taxane with lapatinib-taxane, in the first-line setting of HER2-positive MBC. The lapatinib- taxane combination was found to be inferior for PFS (median, 9.0 vs. 11.3 months; HR = 1.37*P* = 0.001) and there was a trend for OS in favor of the trastuzumab arm.

Several limitations to the current analysis must be noted. The findings are mainly restricted by the nature of a retrospective database review that suffers from patient selection and treatment bias. Potential imbalances in prognostic factors which were not recorded in the CHS database, such as tumor subtype, performance status and dose density and intensity of the chemotherapies combined with trastuzumab cannot be excluded. Besides, missing data on whether any of the patients received new anti HER2 therapies, such as TDM1 or pertuzumab, for compassionate use or as part of a trial, in further therapy lines may affect overall survival and skew the results.

In addition, the outcome endpoint has some weaknesses, as OS is not disease-specific, as described. Since we did not have access to imaging results, we could not retrieve actual PFS data. In some cases treatment was stopped due to toxicity before disease progression, therefore, we could not use the duration of the second-line therapy as a proxy for clinical progression.

However, despite these limitations, we had full information regarding the primary end point with no patients "lost to follow-up". This study in 'real world' practice is important in examining comparative effectiveness of lapatinib and trastuzumab in the context of actual patient care. The results may have impact on decision making in many healthcare settings where the preferred treatment with TDM-1 is not yet available.

## Conclusion

In this retrospective database analysis, derived from actual practice, OS of patients treated with TBP was significantly longer than in patients treated with second-line lapatinib, after controlling for differences in prior brain metastases and rate of previous adjuvant trastuzumab therapy. This study is hypothesis generating and results should be further confirmed by analyses of larger patient populations, preferably in randomized clinical trials.

## Supporting Information

S1 Data Research dataset. (XLSX)
